# Network changes associated with transdiagnostic depressive symptom improvement following cognitive behavioral therapy in MDD and PTSD

**DOI:** 10.1038/s41380-018-0201-7

**Published:** 2018-08-13

**Authors:** Zhen Yang, Shi Gu, Nicolas Honnorat, Kristin A. Linn, Russell T. Shinohara, Irem Aselcioglu, Steven Bruce, Desmond J. Oathes, Christos Davatzikos, Theodore D. Satterthwaite, Danielle S. Bassett, Yvette I. Sheline

**Affiliations:** 1 Center for Neuromodulation in Depression and Stress, Department of Psychiatry, University of Pennsylvania, Philadelphia, PA 19104, USA; 2 Department of Bioengineering, University of Pennsylvania, Philadelphia, PA 19104, USA; 3 Brain and Behavior Laboratory, Department of Psychiatry, University of Pennsylvania, Philadelphia, PA 19104, USA; 4 Department of Radiology, University of Pennsylvania, Philadelphia, PA 19104, USA; 5 Department of Biostatistics, Epidemiology, and Informatics, University of Pennsylvania, Philadelphia, PA 19104, USA; 6 Center for Trauma Recovery, University of Missouri, St. Louis, MO 63121, USA; 7 Department of Electrical and Systems Engineering, University of Pennsylvania, Philadelphia, PA 19104, USA; 8 Department of Neurology, University of Pennsylvania, Philadelphia, PA 19104, USA

## Abstract

Despite widespread use of cognitive behavioral therapy (CBT) in clinical practice, its mechanisms with respect to brain networks remain sparsely described. In this study, we applied tools from graph theory and network science to better understand the transdiagnostic neural mechanisms of this treatment for depression. A sample of 64 subjects was included in a study of network dynamics: 33 patients (15 MDD, 18 PTSD) received longitudinal fMRI resting state scans before and after 12 weeks of CBT. Depression severity was rated on the Montgomery-Asberg Depression Rating Scale (MADRS). Thirtyone healthy controls were included to determine baseline network roles. Univariate and multivariate regression analyses were conducted on the normalized change scores of within- and between-system connectivity and normalized change score of the MADRS. Penalized regression was used to select a sparse set of predictors in a data-driven manner. Univariate analyses showed greater symptom reduction was associated with an increased functional role of the Ventral Attention (VA) system as an incohesive provincial system (decreased between- and decreased within-system connectivity). Multivariate analyses selected between-system connectivity of the VA system as the most prominent feature associated with depression improvement. Observed VA system changes are interesting in light of brain controllability descriptions: attentional control systems, including the VA system, fall on the boundary between-network communities, and facilitate integration or segregation of diverse cognitive systems. Thus, increasing segregation of the VA system following CBT (decreased between-network connectivity) may result in less contribution of emotional attention to cognitive processes, thereby potentially improving cognitive control.

## Introduction

Cognitive behavioral therapy (CBT) is an effective treatment for both major depressive disorder (MDD) and posttraumatic stress disorder (PTSD) with equally efficacious but more enduring effects compared with antidepressants [1–3]. The fact that various mental disorders involving depression can be alleviated by CBT suggests that common neural mechanisms may be engaged in treatment response. However, there are no published studies investigating the network mechanisms involved in transdiagnostic treatment response to CBT common to both MDD and PTSD. Previous studies of brain mechanisms involved in producing this improvement have focused on changes in cognitive control regions in MDD [4–7] and in PTSD [8–11], examined separately, following treatment. Studies examining neural substrates of CBT have demonstrated changes in cognitive control regions following various forms of treatment (reviewed in [4, 7]).

However, network-level effects of CBT are not well understood. Some researchers have applied tools from graph theory and network science to better understand the neural mechanisms of depression treatment, given the increased conceptualization of neuropsychiatric disorders as involving large-scale functional network disorganization [12–17]. Prior research, however, has examined the neurobiological signature of CBT either at a coarse level (i.e., focusing on global topological network features) or at a fine level (i.e., focusing on a specific network). Thus, whether and how the functional interactions between networks within dynamic brain systems contributes to CBT treatment response remains unknown.

A large literature describes a priori network baseline differences in depression, including those in the affective network, the cognitive control network, and the default mode network, as well as interactions between them [18–20]. In addition, abnormalities in the interconnecting structures comprising these systems have been extensively investigated (for reviews see [12, 21–23]). These studies have examined abnormalities using a priori regions of interest but have not conducted data-driven analyses. More recent data-driven studies in depression have examined baseline network properties, including loss of small-world network structure [24] and a significant reorganization of community structure [25–27]. Applications of a few simple metrics from graph theory have provided conflicting results. Some found decreased path length and no change in the clustering coefficient, prominent changes in community structure but no differences in path length and clustering coefficient [25, 26], higher local efficiency and modularity [27] or higher local efficiency and modularity as well as disruptions in the nodal centralities of many brain regions, particularly in the default mode and cognitive control systems [15]. These studies described features of baseline brain network differences in untreated depression; however, they did not examine treatment-associated changes. In the current study, we used network science tools to identify changes in network architecture and function across the treatment time course.

Psychologically, CBT ameliorates depressive symptoms by changing patterns of negative thinking and behaviors [1, 28]. It has been proposed that correcting the imbalanced communication among functional networks plays an important role in the efficacy of CBT treatment responses [29]. With advances in the network neuroscience field, novel tools have been developed that allow a deeper understanding of complex brain functions [30]. Using previously developed tools adapted from studies of airline transportation networks and the Internet [31, 32] demonstrated the emergence of system roles in normative neurodevelopment. Specifically, they showed that development of the functional brain organization is driven by changes in the balance of within- versus between-module (or system) connectivity. These tools allow network roles to be defined based on the position of a module in the two-dimensional plane mapped out by their within- and between-system connectivity. (In the remainder of this paper, we use the term “network” to mean a graph, and “system” to mean a subset of brain regions in order to decrease the potential confusion of using the term “network” to mean both constructs.)

Here, we applied these network neuroscience tools to resting-state fMRI data collected in a transdiagnostic sample (including patients with primary MDD or PTSD) with longitudinal treatment data. The overarching goal was to understand neural correlates of depressive symptom improvement following 12 weeks of manualized CBT treatment across diagnoses. We hypothesized that changes in functional roles of higher-order cognitive networks (thereby putatively decreasing the saliency of emotional signals) would be correlated with changes in depressive symptoms. To confirm the results obtained in network analyses, we also performed a parallel data-driven analysis to select the network features that were most predictive of depressive symptom improvement.

## Methods and materials

### Participants

Our initial sample included 95 participants—64 patients (MDD: *n* = 21; PTSD: *n* = 43) who entered CBT treatment, and 31 healthy controls included to delineate baseline network functional roles. All participants were females, right-handed, English-speaking, and aged 18–55 years. See Table 1 for demographic characteristics. Inclusion diagnosis for MDD and PTSD was established according to DSM-IV-TR [33] and the Clinician Administered PTSD Scale (CAPS) [34]. All PTSD participants had PTSD as the primary diagnosis. PTSD participants had a lifetime mean total score on the CAPS of 78.16 ± 18.51. All PTSD participants reported interpersonal violence-based trauma (rape, domestic violence, etc.); many reported multiple episodes and episodes of longstanding duration. Exclusion criteria included: (1) co-morbid neurological disorders; (2) current alcohol or substance abuse disorder; (3) history of psychotic disorder, bipolar disorder, or obsessive-compulsive disorder; (4) current suicide risk; (5) treatment with any psychotropic or central nervous system-active drug within the previous 3 weeks (5 weeks for fluoxetine). All participants provided written informed consent; the Human Subjects Committees of both Washington University and the University of Missouri-St. Louis approved all study procedures.

The baseline depressive symptom severity and treatment response changes across diagnoses were assessed using the clinician-administered Montgomery-Asberg Depression Rating Scale (MADRS), a scale shown sensitive to symptoms change [35]. Among PTSD participants 21.95% had a MADRS score ≥18. In addition to MADRS, the Anxious Arousal (AA) subscale of the self-report Mood and Anxiety Symptoms Questionnaire (MASQ) [36] was also administered to control for the overlapping effect of anxiety with depression and to test for the specificity of depression-related results. Symptom and brain imaging data were always collected on the same day. For the longitudinal treatment, patients received 12 weeks of manualized psychotherapy, either CBT for MDD or cognitive processing therapy (CPT) for PTSD, delivered or supervised by the same clinical psychologist (SEB), a highly-trained CBT therapist. As defined by the APA Clinical Practice Guidelines (http://www.apa.org/ptsd-guideline/treatments/cognitive-processing-therapy.aspx): CPT is a specific type of CBT that has been effective in reducing symptoms that have developed after experiencing a variety of traumatic events [9, 37–39].

For this dataset, we checked data quality before performing any statistical analyses. Following data exclusions (see Supplement), a longitudinal imaging sample of 15 patients with MDD and 18 patients with PTSD with usable MRI scans at two time points was included. PTSD participants were treated at the Center for Trauma Recovery, University of Missouri-St. Louis, and MDD participants were treated at Washington University, St. Louis. All participants received their MRI scans at Washington University. Patients (*n* = 33) and controls (*n* = 31) did not differ in age (*p* > 0.20), though patients had significantly lower education levels than controls [16.79 ± 2.19 versus 15.39 ± 1.62; *p* = 0.01]. No patients were on current psychotropics. Demographic information is available in the Supplement.

### Imaging data acquisition and preprocessing

Imaging was performed for all subjects on the same scanner (Siemens 3T Trio) using the same acquisition protocol (see Supplement). Preprocessing details of the T1 images are described elsewhere [40]. Resting-state time series data were processed using a validated confound regression procedure optimized to reduce the influence of subject motion [41]. See the Supplement for further details about fMRI preprocessing and motion correction.

### Functional network construction

**Fig. 1:**
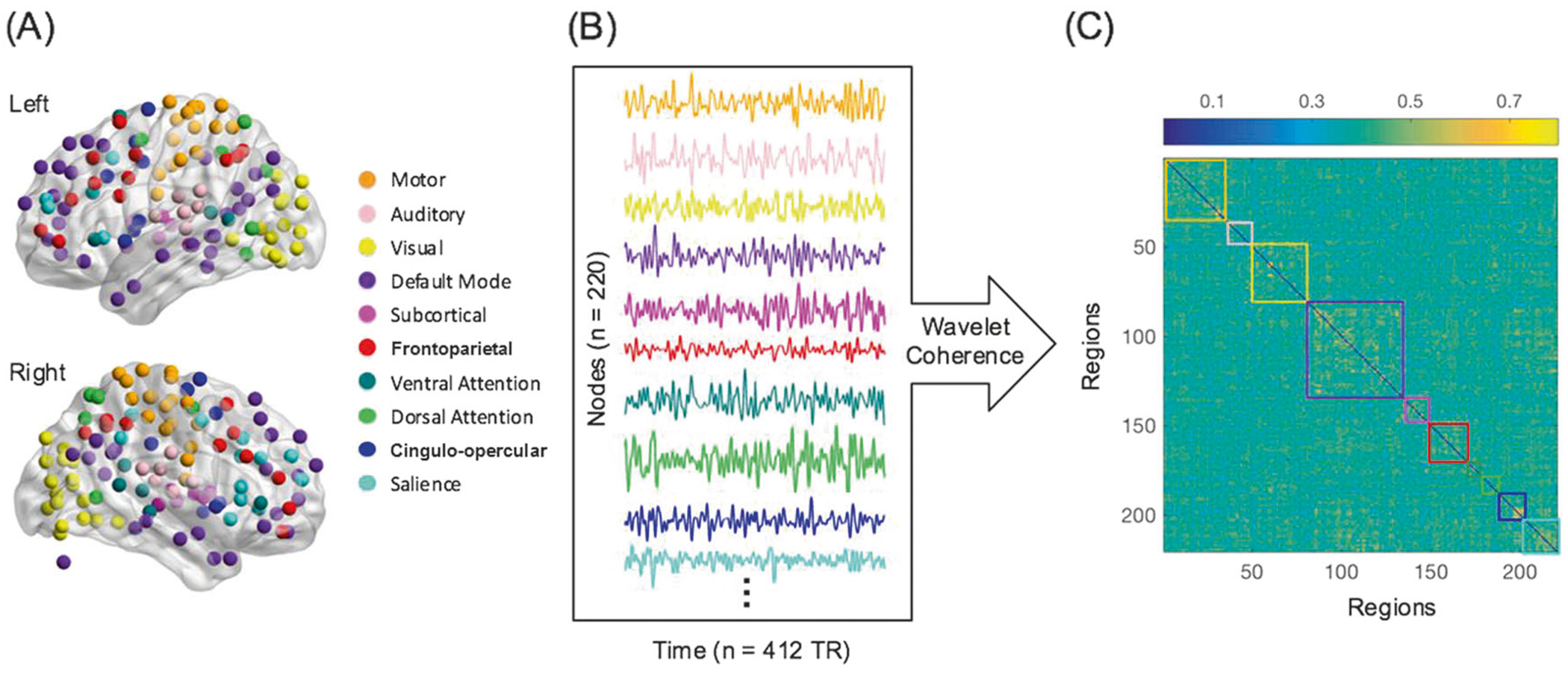
Functional network construction. The nodes we used were defined in a separate adult sample (**a**: [42]). Functional connectivity was estimated by applying a wavelet coherence to the ROI mean BOLD signals between every pair of nodes in the range of 0.01–0.08 Hz. BOLD signal time series and the set of pairwise coherence values were shown in **b, c** for an exemplar subject

The nodes included were functionally defined in a separate adult sample [42]. The original parcellation included 264 ROIs. To ensure the quality and interpretability of data, we excluded 7 ROIs with poor coverage and 28 ROIs not assigned to a specific network. To increase the stability of network measures, we excluded small systems with ≤5 ROIs (cerebellum: *n* = 4 and memory retrieval: *n* = 5) (see Supplement Methods and Figure S1 for justification of this threshold. See Supplement Results for stability analyses). We further combined somatomotor mouth (*n* = 5) with somatomotor hand into one motor network. Thus, our final analyses included 220 nodes that belonged to 10 systems. Functional connectivity between these 220 ROIs was estimated using wavelet coherence in the typical low-frequency range used for resting state data: 0.01–0.08 Hz (Fig. 1). A wavelet-based method was utilized based on previous work introducing the approach of determining module roles within the network [43] due to its advantages over the Pearson’s correlation coefficient [25] (see Supplement for further details).

For each subject, the functional brain network was depicted by a fully weighted adjacency matrix in which network nodes represented brain regions and network edges represented functional connections between those regions. To explore the full connectivity patterns related to mood and anxiety disorders, we did not apply any arbitrary thresholds to the functional connectivity matrix. To control for residual effects of motion and other global effects unaccounted for during preprocessing, we divided each subject’s functional connectivity matrix by its average value to obtain the normalized functional connectivity matrix [44], which was used for the remainder of our analysis.

### Roles of intrinsic functional networks

**Fig. 2:**
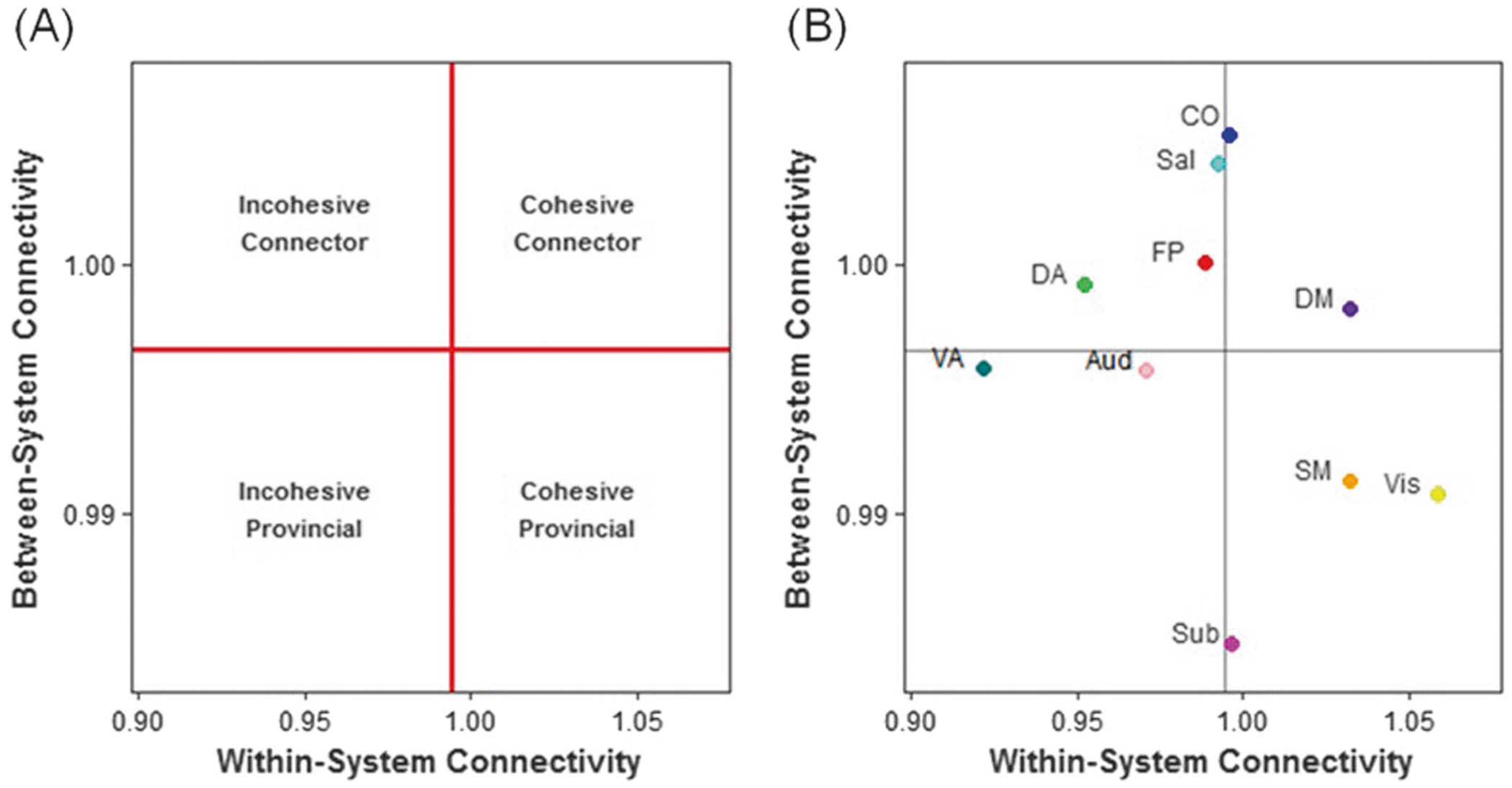
System functional roles in healthy controls in the current sample. The two-dimensional space mapped out by the within- and between-system connectivity were divided into four quadrants **(a)**. The lines demarcating the boundaries of the quadrants are defined by the average within- and average between-system connectivity across all systems. A system with high within- and high-between-system connectivity was defined as cohesive connector; a system with high within- and low-between-system connectivity was defined as a cohesive provincial; a system with low within- and high-between-system connectivity was defined as incohesive connector; and a system with low within- and low-between-system connectivity was defined as incohesive provincial. **b** displays the system functional roles of the current healthy control sample (see Table S1 for within- and between-system connectivity of each system)

The functional role of each network depends on two properties: the within-system connectivity and the between-system connectivity [32]. Mathematically, the within-system connectivity is defined as: $R_{i}=\frac{\sum_{i,j\in C_{i}}{\tilde{\text{A}}}_{ij}}{{\left|C_{i}\right|}^{2}}$. Between-system connectivity is defined as: $I_{i}=\frac{\sum_{i\in C_{i},j\notin C_{i}}{\tilde{\text{A}}}_{ij}}{\left|C_{i}\right|\cdot \left(N-\left|C_{i}\right|\right)}$ Here $\tilde{\text{A}}$ is the weighted adjacency matrix normalized by its mean, $\left|{\text{C}}_{i}\right|$ is the size of the *i*th community, and *N* is the number of nodes in total. These two measurements quantify the average strength of the within- and between-system connectivity, producing a two-dimensional space that can be divided into four quadrants or categories (Fig. 2a): cohesive connector, cohesive provincial, incohesive connector, and incohesive provincial [32]. The line that differentiates cohesive systems from incohesive systems represents the mean within-system connectivity across all systems. The line that differentiates connector systems from provincial systems represents the mean between-system connectivity across all systems. Based on these definitions, each system falls into one of the following four categories (Fig. 2a): cohesive connector, cohesive provincial, incohesive connector, and incohesive provincial [32]. No system exists in more than one category.

### Univariate analyses

To examine the neural correlates underlying depressive symptom improvement following CBT, we examined how changes in depressive symptoms relate to changes in network roles of each system. Both change in MADRS and change in network features (within- or between-system connectivity) were quantified using a normalized score defined as 100 × (postCBT-preCBT)/preCBT, yielding a percent change. We performed partial correlations and included age and head motion (quantified using mean framewise displacement) as covariates. The network role of a given system was defined in a two-dimensional space (both within- and between-system connectivity). We determined the significance of the partial correlation in two dimensions using a two-dimensional permutation test [32] and determined each dimension separately using a non-parametric bootstrap approach. For the bootstrap, we first generated 1000 bootstrap sample by sampling 15 MDD and 18 PTSD patients with replacement from the original data for each system. We then re-computed the partial correlation on the bootstrapped sample to obtain a 95% confidence interval. Multiple comparisons were adjusted for using the false discovery rate (FDR) correction at a threshold of *q* = 0.05. Specifically, we corrected for 10 tests for two-dimensional analyses (one test/system) and for 20 tests for one-dimensional analyses (10 tests for within-system connectivity and 10 tests for between-system connectivity).

### Specificity analyses

Because changes in depression and anxiety symptoms were significantly correlated (*r* = 0.68, *p* < 0.001), we repeated the above analyses by including changes in anxious arousal (MASQ-AA score) as a covariate in addition to age and head motion to test for the specificity of depressive symptom-related results.

### Multivariate analyses

In univariate analyses, we examined the brain-symptom association for each system. Here, we confirmed the roles of these systems by performing a multivariate regression analysis. Specifically, the normalized change scores of within- and between-system connectivity of the ten systems together with the change scores of the in-scanner head motion were included as predictors. This process resulted in 21 predictors. The dependent variable was the normalized change in MADRS score. Age was linearly regressed out from each predictor and dependent variable before regression modeling. We used linear regression with an elastic net penalty [42, 45], which shrinks and selects a sparse set of predictors. The elastic net penalty is a combination of the LASSO penalty [46], which induces sparsity and a ridge regression penalty [47], which reduces multicollinearity among predictors. A grid search was conducted across potential values of the penalty tuning parameters. The optimal tuning parameters were selected by cross-validation, as detailed further in the Supplement (Figure S2 and S3).

## Results

### Roles of intrinsic functional systems in healthy controls

Before examining whether system role changes following CBT were related to depression improvement, we first defined the average functional roles of each system in healthy controls. (see Fig. 2b, Table S1 for baseline functional role delineation). We note that the relative location of these systems is very similar to what was observed in a previous study using the same method [32].

### Brain-symptom association following CBT: univariate analyses

**Fig. 3:**
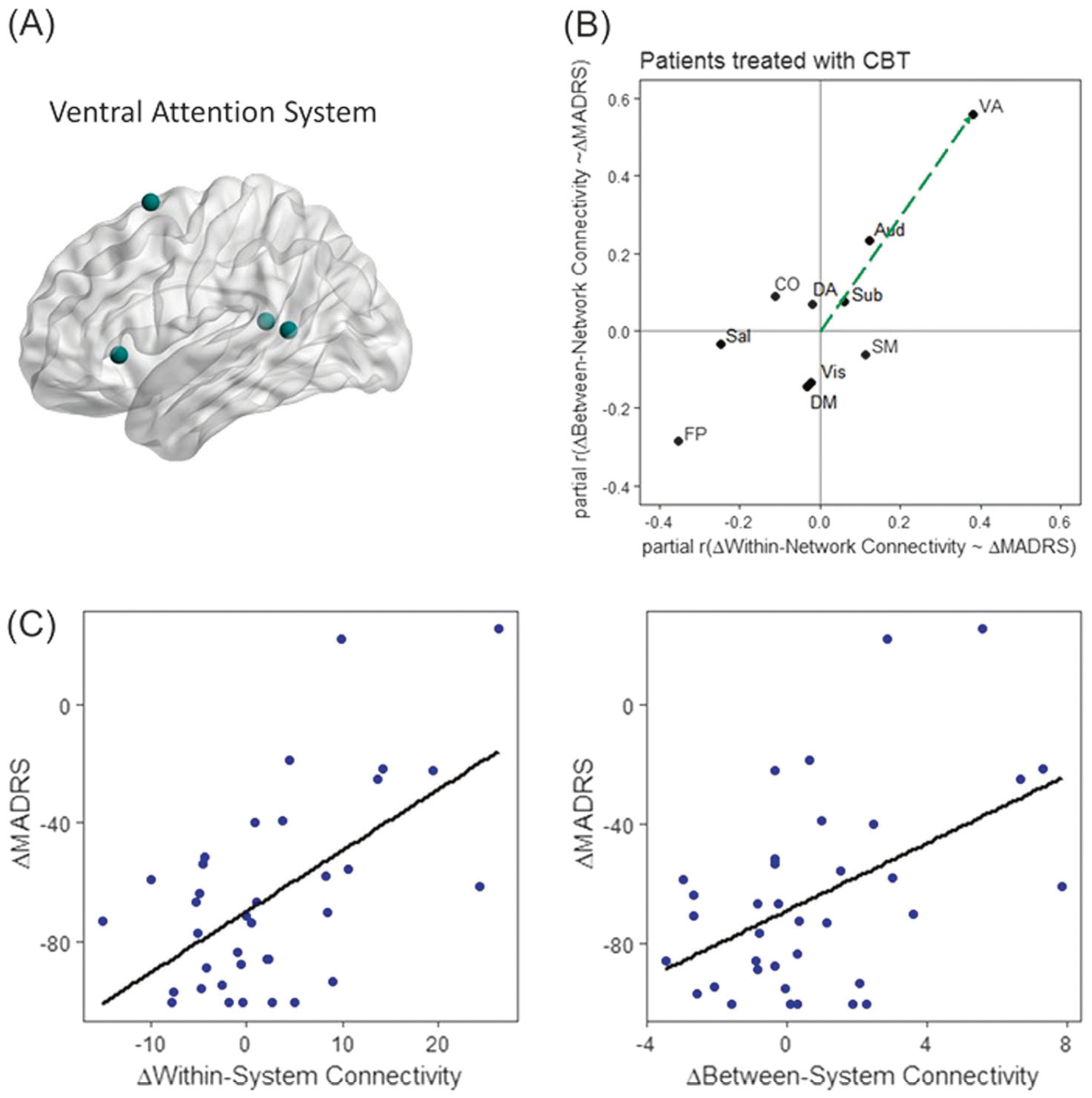
The degree of depressive symptom improvement was associated with the level of changes in system functional roles of the ventral attention (VA) system. The spatial location of nodes belonging to the VA system were shown on the left lateral view in **a**. Following CBT, the VA system functional role change was significantly associated with the changes in depressive symptoms measured using MADRS after controlling for age and head motion **(b)**. Specifically, greater improvement in depressive symptoms was associated with greater decrease in both within- and between-system connectivity of the VA system **(c)**. The VA system functions as an incohesive provincial. Our longitudinal results indicate that further increase in the functional role of the VA system as an incohesive provincial (less within- and less between-network connectivity after CBT) is associated with greater symptom amelioration

Paired sample *t*-tests revealed that 12 weeks of CBT treatment significantly improved patients’ depressive symptoms assessed by MADRS [*t*_(32)_ = −7.45; *p* < 0.001, *d*= 1.68; mean reduction relative to baseline: 64.15%], and anxious arousal symptoms assessed using MASQ-AA [*t*_(23)_ = −2.98, *p* = 0.007, *d* = 0.88; mean reduction: 18.02%]. When we correlated system functional roles with symptom changes we found that greater symptomatic improvement in depression was associated with a greater increase in the functional role of the VA system as an incoherent provincial system (two-dimensional permutation test: *p* = 0.005, FDR corrected). This effect is mainly driven by the decrease in between-system connectivity (partial *r* = 0.71; *p* < 0.001, FDR corrected, 95% confidence interval: [0.53, 0.86]). Decrease in the within-system connectivity approached significance (partial *r* = 0.49; *p* = 0.005; 95% confidence interval: [0.09, 0.77]) (Fig. 3). Our specificity analyses suggest that this brain–symptom association is specific to depressive symptoms (see Supplement for detailed results). No other systems showed significant associations with symptom changes.

To better interpret our key finding that the changes in depressive symptoms (normalized by baseline) were significantly correlated with the changes in functional role of the VA system in patients, we examined the baseline connectivity differences between patients and controls using a two-dimensional significance test. We found that the distance between patients and controls in the two-dimensional space is not significant in any of the systems after FDR correction (Figure S4). We also found that the overall VA system functional role change following CBT is not significant at the group level (*p* > 0.1). However, the changes in between-system connectivity of the VA system significantly differed between patients and controls [*t*(52) = 2.25, *p* = 0.03, *d* = 0.62], and marginally differed in VA within-system connectivity [*t*(52) = 1.71, *p* = 0.09, *d* = 0.49, suggesting that the connectivity change in patients following CBT has an association with CBT.

### Brain–symptom association following CBT: multivariate analyses

**Fig. 4:**
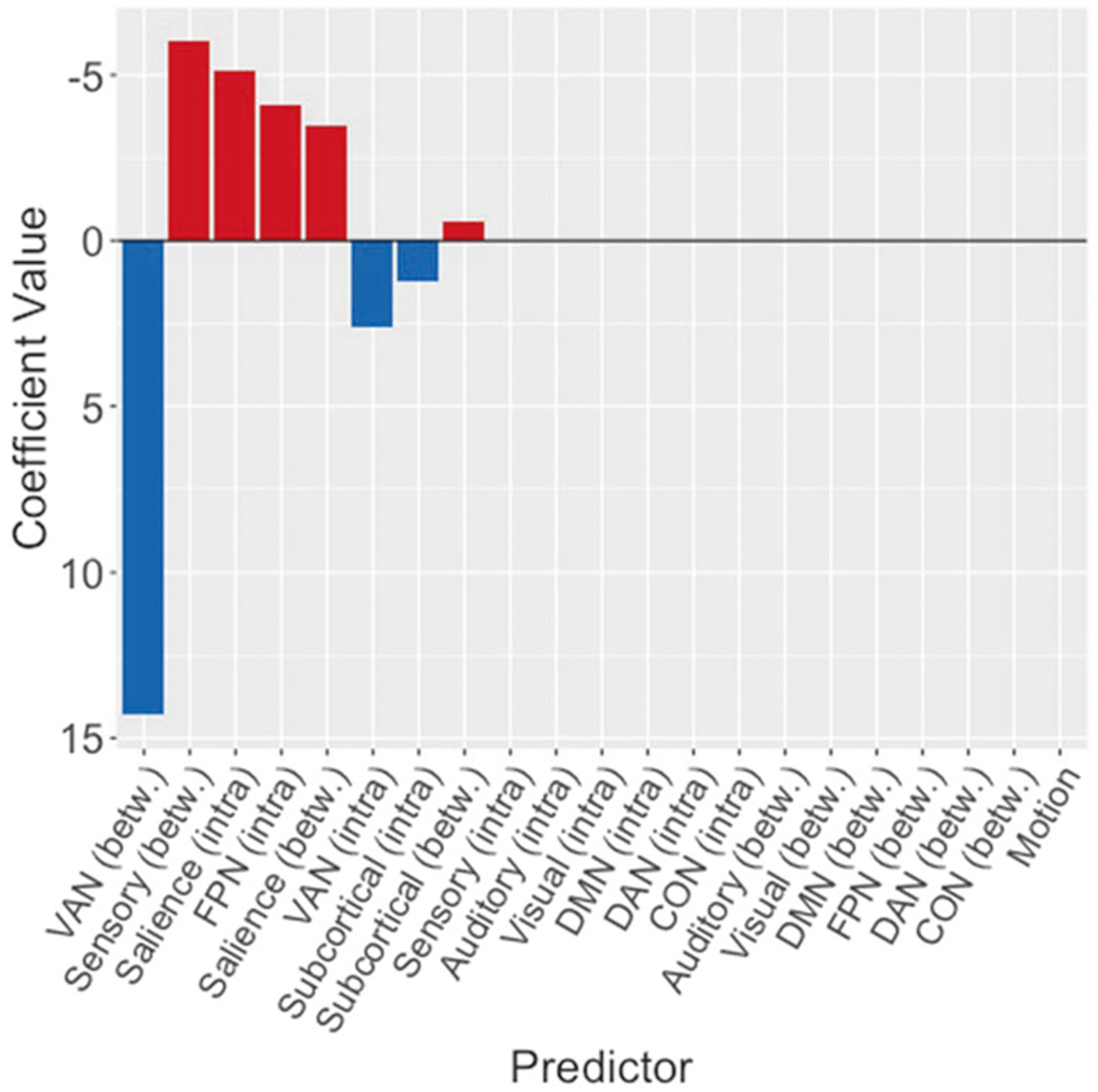
Model coefficients of predictors selected by the optimal (cross-validated) elastic net regression. The magnitude of VA between-system connectivity is much greater than all other predictors. The direction of the coefficient indicates that greater improvement in depressive symptoms was associated with greater decrease in between-system connectivity of the VA network

The coefficients obtained from the elastic net regression are shown in Fig. 4. To assess the significance of these associations, we applied the Covariance test [48] which provides inference that accounts for variable selection performed by the elastic net. Following application of the Covariance test, decreased between-system connectivity of the VA system was the only variable significantly associated with symptom improvement following CBT. Additional results, which are provided in the Supplement (see Supplement Results, Figure S5, S6, and S7), showed non-significant associations with CBT outcome. Finally, we note that the coefficient associated with motion was near zero and non-significant according to the Covariance test, suggesting that in-scanner motion did not corrupt our results.

## Discussion

Here we examined the effect of CBT on network organization to determine how the functional interaction between functional systems contributes to CBT treatment response. By extracting system-level network features for analysis, we aimed to facilitate biological interpretability while reducing the dimensionality of the imaging data more dramatically than a region-level analysis would have acheived. Based on the balance of within- vs. between-module connectivity, this integrative approach maps the functional role in a two-dimensional space and characterizes how a system interacts with other systems. We find that the VA system tends to be an incohesive provincial system at baseline and its functional role does not differ between patients and controls. However, individual differences in the change in functional role of this system are behaviorally relevant and correlated with the degree of symptom improvement. Using a data-driven analysis, we determined a primary association between decreases in between-system connectivity of the VA system and reductions in MADRS following CBT. The multivariate results were in agreement with the univariate analysis, corroborating the key role of decreased VA between-system connectivity in CBT treatment response. This is in keeping with the known role of the VA system in detecting higher-order salient stimuli [49], either subsuming or overlapping with the salience system [50] and suggesting that what is “salient” changes with treatment. These results suggest that increased segregation of this network from other systems may play an important role in the treatment effect. Given the lack of baseline difference, the relationship may reflect a compensatory rather than a restorative mechanism.

One reason for interest in resting-state fMRI functional connectivity (rs-FC) and techniques to probe its fundamental properties is the potential to identify a universal intrinsic network architecture present across brain states. Meta-analytic task activation studies have related task activation patterns to rs-FC [51]. Interrogating a variety of individual task states, other studies [52] found that the actual task architectures (after regressing out task effects) were very similar to the rs-FC architecture. While approximately 25% of connections would still be changed during task performance [49], rs-FC reflects the standard state of the brain’s functional networks, common across task performance, and provides an understanding of the brain’s functional organization across a wide variety of brain states.

Our findings of primary changes in the VA system are interesting in light of a study describing brain structural controllability. That study [43] found that attentional control systems, including the VA system, fall on the boundary between-network communities and facilitate the integration or segregation of diverse cognitive systems. In the current study, the primary system realignment was a change in the VA system towards increased segregation (e.g., decreased between-system connectivity). By producing more system segration, the resulting network relationships, including those with the salience and subcortical systems, were more isolated from attention to emotion, which could potentially diminish the effect of low mood, anhedonia, irritability, and rumination. Consistent with the idea of a baseline over-active VA system [53], this system has been linked to anxiety disorders, with increased stimulus-driven attention, especially for emotionally-laden stimuli. Among children with a history of anxiety or depression, the magnitude of the VA system rs-FC was correlated with measures of attention bias towards threat [54]. CBT has been shown to significantly decrease bias towards threat [55], in keeping with addressing an over-active VA system.

The multivariate results presented in this paper were obtained by performing elastic net penalized regression [45]. Elastic net regression has been increasingly used in neuroimaging and psychiatry [56], in particular, to determine what brain regions are impacted by depression [57] and to predict clinical outcomes [58, 59]. The findings of the multivariate analysis are also important to understanding how CBT-related decrease of depression symptoms might work, since it allows potential simultaneous contributions from multiple systems. In that analysis, the VA between-system connectivity was by far the strongest predictor of symptom changes (and the only significant predictor following application of the Covariance Test), validating the results of the univariate analysis.

### Limitations

We note that our PTSD participants were all intimate partner violence survivors and our results may not generalize to other PTSD populations. Further studies will be necessary to explore the effects of population heterogeneity on network changes in brain–behavior relationships. Furthermore, the lack of an active control group limits our interpretation of network changes as purely CBT-induced. Other factors such as general clinician contact or illness progression may also contribute to the changes in VA network connectivity following treatment. However, changes in between-system connectivity of the VA system in patients significantly differed from controls, suggesting these changes may related to CBT.

Another limitation is that the illness duration and history of psychotropic medication were not recorded for our patient group, although no patients were actively taking medication during the study. Future investigations that include these data will help to determine the potential impact on treatment effect. Finally, our sample size is relatively small and included heterogeneous patients, which limited our power to conduct diagnosis specific analyses, especially for small effect sizes. However, our transdiagnostic approach allows us to investigate the shared neural substrate underlying CBT across MDD and PTSD. It is essential for future work to replicate the current results with a larger and more homogeneous sample.

### Conclusions

We identified a common transdiagnostic change in network structure underlying change in depressive symptoms across MDD and PTSD. Our results demonstrate changes in common across DSM categories following CBT and support the utility of a dimensional approach to identifying treatment-associated brain–behavior links.

## Supplementary Material

supplement

The online version of this article (https://doi.org/10.1038/s41380-018-0201-7) contains supplementary material, which is available to authorized users.

## Figures and Tables

**Table 1:** Sample characteristics

	Initial sample (*n*=95)			Longitudinal subsample (*n*=54)		
	HC	Patients		HC	Patients	
		MDD	PTSD		MDD	PTSD
Sample size	31	21	43	21	15	18
Age (SD)	32.7 (9.32)	33.0 (8.73)	31.2 (10.1)	31.6 (9.87)	32.9 (7.71)	31.6 (11.1)
Education (SD)	16.7 (2.33)	15.0 (2.14)	15.1 (1.88)	17.1 (2.45)	15.4 (1.64)	15.4 (1.65)
MADRS (SD)	1.19 (1.91)	27.7 (6.38)	16.7 (8.64)	1.25 (2.55)	8.27 (7.66)	5.11 (4.50)
MASQ-AA (SD)	19.8 (3.36)	35.3 (12.3)	33.6 (9.92)	19.0 (2.68)	26.6 (9.45)	23.0 (5.76)
CAPS_m (SD)	N/A	N/A	69.1 (17.3)	N/A	N/A	19.2 (15.0)

*MADRS* Montgomery-Asberg Depression Rating Scale, *MASQ*-AA Anxious Arousal subscale of Mood and Anxiety Symptoms Questionnaire, *CAPS*_m Clinician-Administered *PTSD* Scale past-month total. *HC* healthy control subjects, *MDD* patients with major depressive disorder, *PTSD* patients with posttraumatic stress disorder, *SD* standard deviation, *N/A* not applicable
