# Supplementary material for: Network changes associated with transdiagnostic depressive symptom improvement following cognitive behavioral therapy in MDD and PTSD
Source: Mol Psychiatry. Author manuscript; Available in PMC 2026 Apr 27. (PMC13112374; doi:10.1038/s41380-018-0201-7)
Supplement: supplement [file NIHMS2166283-supplement-supplement.docx]

**Supplemental Materials**

**Methods**

*Data exclusions*

Among the initial sample, 17 MDD and 27 PTSD participants completed treatment; 16 MDD and 22 PTSD patients returned for followup imaging. Among those who completed the CBT treatment and had imaging data, four PTSD and one MDD patients were further excluded. For PTSD patients, two were missing MADRS scores after treatment, one was an outlier in changes in MADRS score (outside of 3 inter-quartile range), and one had high head motion (mean framewise displacement outside of 3 inter-quartile range of all subjects). The MDD patient was excluded due to high head motion. Among the intial sample, 24 controls returned for the 12-week followup scan. three subjects were excluded: two due to high head motion and one due to poor image coverage (over 5% of the nodes were outside of the brain). We also visually inspected the imaging registration and no outliers were found.

*Check for Sampling Bias*

To assess potential sampling bias and assure the generalizability of our results, we compared demographics (age, sex, and education level), clinical symptoms, and brain network measures between the patients who completed 12 weeks of manualized CBT treatment and had usable MRI scans at both time points (n=33) and patients who did not complete CBT (n=31). We found that the two patient groups did not differ in age, sex, or years of education (p > 0.10); severity of depressive and anxiety symptoms (p > 0.10); or within- and between-system connectivity of the VA system (p > 0.05).

*Imaging parameters*

High-resolution structural images were acquired using a T1-weighted MPRAGE sequence: TR 2400 ms, TE 3.13 ms, TI 1000ms, flip angle 8°, slice thickness/gap 1mm/0mm, effective voxel resolution 1.0mm3. Resting-state gradient spin-echo functional images were acquired in two series of 210 volumes (7:42 duration each) using the following parameters: TR 2200 ms, TE 27 ms, flip angle 90°, slice thickness/gap 4mm/0mm, effective voxel resolution 4.0mm^3^.

*fMRI Preprocessing and Motion Correction*

After the first 4 volumes of the functional timeseries were removed to allow signal stabilization, functional images were re-aligned using MCFLIRT [^1^](#_ENREF_1) and smoothed with a Gaussian filter at 6mm FWHM. Confound regression included 9 confounding signals (6 motion parameters + global / WM / CSF) as well as the temporal derivative, quadratic term, and temporal derivative of the quadratic of each (36 regressors total) ^[2](#_ENREF_2)^. Finally, timeseries were band-pass filtered to retain frequencies between 0.01-0.08 Hz; all motion parameters and confound timecourses were band-pass filtered in an identical fashion as the timeseries data itself in order to prevent frequency mismatch [^3^](#_ENREF_3). Functional images were co-registered to the T1 image using boundary-based registration [^4^](#_ENREF_4) and aligned to template space [^5^](#_ENREF_5). Briefly, we first created a custom template with ANTs [^6^](#_ENREF_6) to avoid registration bias and maximize sensitivity to detect regional effects that can be impacted by registration error. We then normalized the T1 images to this study-specific template using SyN diffeomorphic registration which is implemented in ANTs ^[7](#_ENREF_7)^. Next, this custom template was used to guide brain extraction, N4 bias correction [^8^](#_ENREF_8), and Atropos probabilistic tissue segmentation [^6^](#_ENREF_6). Structural image processing was implemented in the ‘antsCorticalThickness’ function [^9^](#_ENREF_9). Throughout, all transformations were concatenated so that only one interpolation was performed.

*Selection of 5ROIs as a threshold*

We selected this threshold for two reasons: (1) The two networks with 5 or 4 nodes lie at the tail in our plot showing the system size sorted in descending order (**Figure S1**) . There is a large gap between these two systems and the systems before them in terms of community size, motivating our use of size 5 as a reasonable cut off. (2) Based on our criteria (≤5 ROIs), cerebellum and memory retrieval were identified as systems that will be excluded. These two networks are a non-cerebral system and a system whose function is less well characterized (it has no correspondence to previously known functional systems: ^[10](#_ENREF_10)^. To ensure our results were stable, we also repeated our analyses without applying any network threshold, that is, including all 12 systems. See Results below.

*Rationale for Wavelet Methodology*

Unlike Pearson’s correlation, wavelet coherence is less sensitive to outliers [^11^](#_ENREF_11) and more sensitive to statistical similarities in the selected power spectra of the two BOLD activity traces. A wavelet coherence is given by a ratio of the cross-spectral density between the first and the second time series, and the product of the autospectral density of these two time series ^[12](#_ENREF_12)^. It provides a broader estimation of functional connectivity [^13^](#_ENREF_13) and offers a fairer assessment of the magnitudes of time series similarity that is independent of inter-regional differences in hemodynamic response function. Pearson’s correlation coefficient can be affected by inter-regional differences in the hemodynamic response function which may cause variations that are independent of the underlying neural activity. With a maximum value of 1 and a minimum value of 0, wavelet coherence is better adapted to the parsimony of network modeling methods that deal with positive-only edge weights [^14^](#_ENREF_14)^,^ [^15^](#_ENREF_15).

*Multivariate Analyses:Elastic Net Parameter Selection*

The elastic net incorporates two penalty terms into a regression analysis. One penalty corresponds to the LASSO, a penalized regression model that induces sparsity by forcing weak regression coefficients in the model to zero as they are more heavily penalized. Forcing coefficients to zero is equivalent to dropping those terms from the model. The second penality, called the ridge penalty, shrinks all coefficient estimates towards zero but without penalizing them completely to zero. There are many possible values that these two penalties may take, and thus they are often referred to as tuning parameters. Often, the optimal tuning parameters are chosen using cross-validation, which was the approach we took in our analysis. We selected the elastic net parameters using a grid search as follows. For 35 values of the ridge penalty ranging from 0.01 and 100000 (more specifically, the sequence 10^x^, where x took all integer values from -2 to 5), we performed ten-fold cross-validation to select the optimal LASSO parameter for that specific ridge parameter.

**Figure S2** presents the best cross-validation errors obtained for each value of the ridge penalty grid search. The best (minimal) error was obtained for a ridge penalty of 0.25. In the software that implements the elastic net regression, the LASSO pentalty is re-parameterized in terms of the overall magnitude of the coefficient vector, specifically, its L_1_ norm. A grid search is then performed over many possible values of the L_1_ norm, scaled by the maximum L_1_ norm so that the grid search is performed over the range [0, 1]. We refer to grid values in this range as the normalized LASSO penalty. **Figure S3** shows all the cross-validation errors obtained for a ridge penalty of 0.25 when the normalized LASSO penalty is increased. The optimum was reached for a normalized LASSO penalty close to 0.2.

**Results**

*Test for stability of Analyses*

To test whether our results were sensitive to the threshold we used to select networks, we repeated our primary analyses but included all 12 systems. Without applying any threshold to select systems, we found that the VA between-system connectivity results remained significant after including the two smaller systems (memory retrieval and cerebellum).

*Baseline functional role delineation in controls*

We found that three high-order cognitive systems (i.e., the frontoparietal, dorsal attention, and salience system) showed high between-system and low within-system connectivity, indicating their roles as incohesive connectors. Cingulo-opercular system shows high between and high within-system connectivity, indicating its role as a cohensive connector. Ventral attention and auditory system have low within and low between-system connectivity and functioned as an incohesive provincial system. Default mode system lies in the cohesive connector quadrant. Somatomotor, visual, and subcortical system lie in the cohesive provincial quadrant (**Figure 2B, Table S1**).

*Specificity Analyses*

When changes in anxiety symptoms measured using MASQ-AA score were also included in the model we found that the original brain-symptom association remained significant for between-system connectivity (partial *r* = 0.67, [0.32, 0.89]) and for within-system connectivity (partial *r* = 0.57, 95% confidence interval: [0.004, 0.88]) for the VA system. This finding suggests that this brain-symptom association is specific to depressive symptoms.

*Significant predictors*

**Figure S5** presents the full coefficient path of the predictors for a ridge penalty of 0.25. For the optimal normalized LASSO penalty of 0.2, decreases in connectivity of the VA system and the within-system connectivity of the subcortical regions are associated with a decrease in clinical symptoms. That is, these variables are included in the optimally penalized model and have positive estimated coefficient values. By contrast, decreases in within-connectivity of the frontoparietal system, and connectivity between the sensory system and the rest of the brain are associated with a less successful CBT outcome. That is, these coefficients are also included in the optimal model and have negative estimated coefficient values.

We used the covariance test [^16^](#_ENREF_16) to assess significance of the predictors in the elastic net models. For a given ridge regression tuning parameter, the covariance test generates a test statistic for each predictor which can be compared to its known null distribution. **Figure S6** presents the predictors selected by the elastic net for a range of ridge regression tuning parameters. We note that the tuning parameters have been re-parameterized so that small values correspond to the most sparse models and larger values correspond to the least sparse models. For small values of the ridge tuning parameter, determined to be optimal by cross-validation (and thus presented in the main text), the between-system connectivity of the VA system was the only predictor selected, and thus the elastic net prediction was primarily based on this single predictor in the regression model. For large values of the ridge tuning parameter, four features were selected according to the covariance test for predicting MADRS change scores. This set of features included the within- and between-system connectivity of the VA system, within-network connectivity of the salience system, and between-network connectivity of the frontoparietal system.

*Jackknife stability*

We studied robustness of the previous results using the jackknife ^[17-19](#_ENREF_17)^. More precisely, we generated jackknife samples by dropping the subjects of our dataset one by one, and we re-computed all the covariance test statistics for each jackknife sample. We obtained for each of the twenty-one predictors thirty-three “jackknife replicates” of the curve shown in the **Figure S7**, where each curve corresponds to the removal of a single subject.

These test statistics, which follow a Fisher-Snedecor distribution with (2,11) degrees of freedom, were transformed into statistics corresponding to the Fisher-Snedecor distribution with (2, 12) degrees of freedom to allow the comparison with the results presented in Figure S6. Then, for each predictor we computed the median of the thirty-three curves, which corresponds to a robust jackknife estimate of the corresponding curve in **Figure S6**. **Figure S7** shows that the robust jackknife results are in agreement with the prior results, which demonstrates that our results are stable with respect to dataset perturbations.

**Figures**

**Figure S1.** Number of ROIs included in each system sorted in descending order.


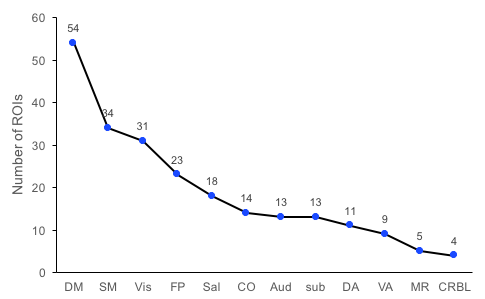


**Figure S2.** Average best cross-validation errors (black line) obtained from the LASSO penalty optimization for a grid of potential ridge regression penalties. Red lines show one standard deviation (pointwise) on either side of the black line.
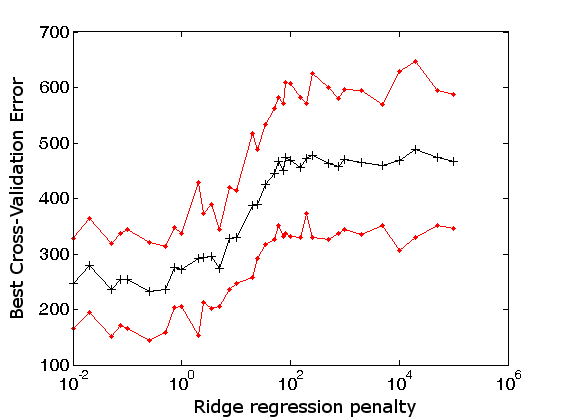


**Figure S3.** Cross-validated mean squared error (MSE) error for a range of normalized LASSO penalties with a fixed ridge penalty of 0.25. The optimum is obtained for a normalized LASSO penalty close to 0.2.


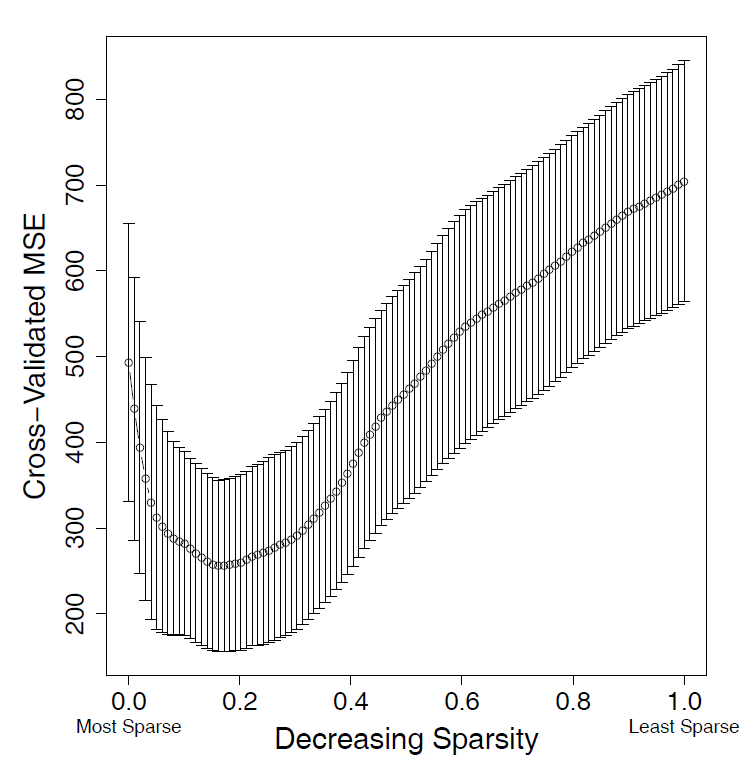


**Figure S4.** functional connectivity profiles of patients and controls plotted in 2-dimensional space. Functional role demarcations were the same as shown in **Figure 2B** which were defined based on controls.

*
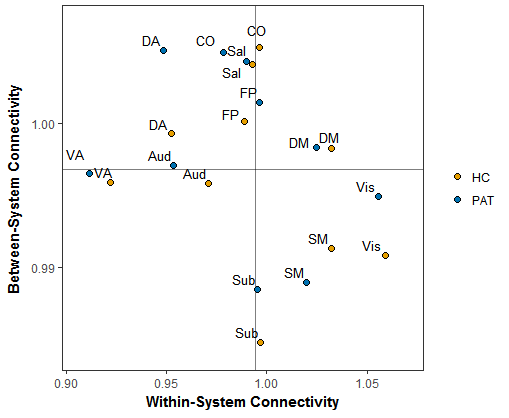
*

**Figure S5.** Regression coefficient predictors as a function of the LASSO penalty for the elastic net using a fixed ridge penalty of 0.25. The x-axis shows an increasingly normalized LASSO parameter, which corresponds to a decreasing penalty on the parameter vector, allowing more predictors to be chosen for the model. For a normalized LASSO of 0.2, both within- and between-connectivity of the VA system and within-connectivity of the subcortical system are selected with a positive weight. Predictors selected with a negative weight include the within-connectivity of the salience, frontoparietal, and visual systems and the between-connectivity of the salience, subcortical, and sensory systems. Key: **DM**: default mode; **FP**: fronto-parietal; **VA**: ventral attention; **DA**: dorsal attention; **CO**: cingulo-opercular. **Intra**: within-system connectivity defined as the average functional connectivity between pairs of nodes within each system; **betw**: between-network connectivity defined as the average functional connectivity between each node of this system with all other nodes outside of this system.


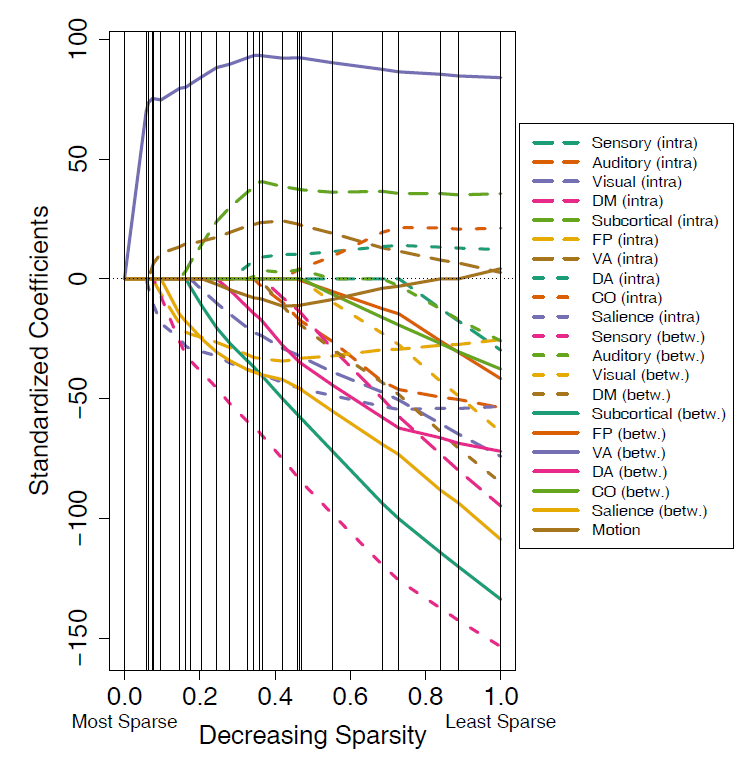


**Figure S6.** The covariance test statistic associated with the network features considered as potential predictors in a model for change in MADRS, plotted for a range of ridge regression tuning parameters. Note that smaller (larger) values on the x-axis correspond to sparser (less sparse) models. These test statistics follow a Fisher-Snedecor distribution witt [^2^](#_ENREF_2)^,^ [^13^](#_ENREF_13) degrees of freedom. The dotted line corresponds to a significance level of 0.001, which corresponds approximately to a Bonferroni correction for 21 tests. The five predictors reaching this significance level for at least one penalty value are displayed in bold.


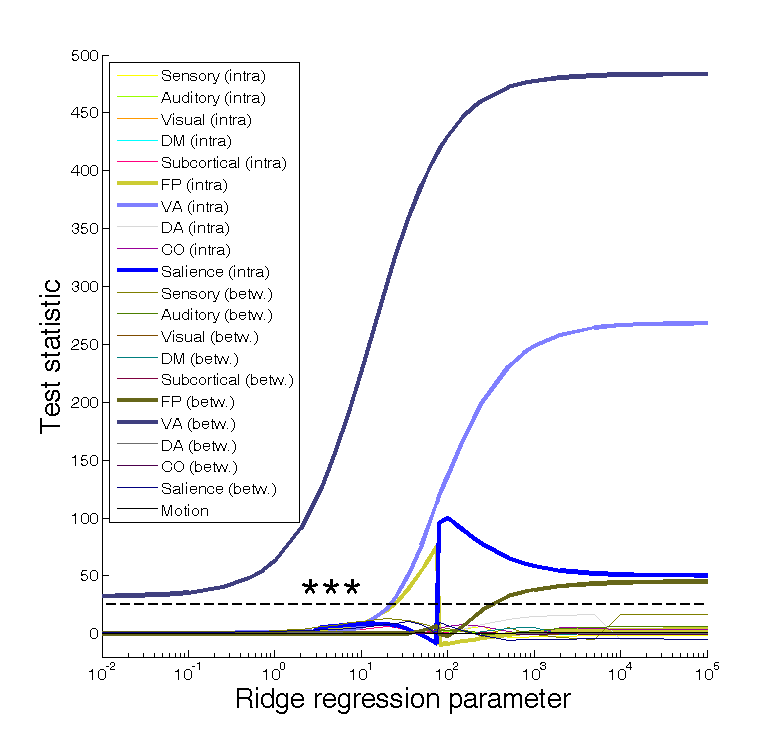


**Figure S7.** Robust jackknife estimation of the covariance test statistics, for each of the 21 network predictors. These results are in agreement with significance results presented in **Figure S3**.


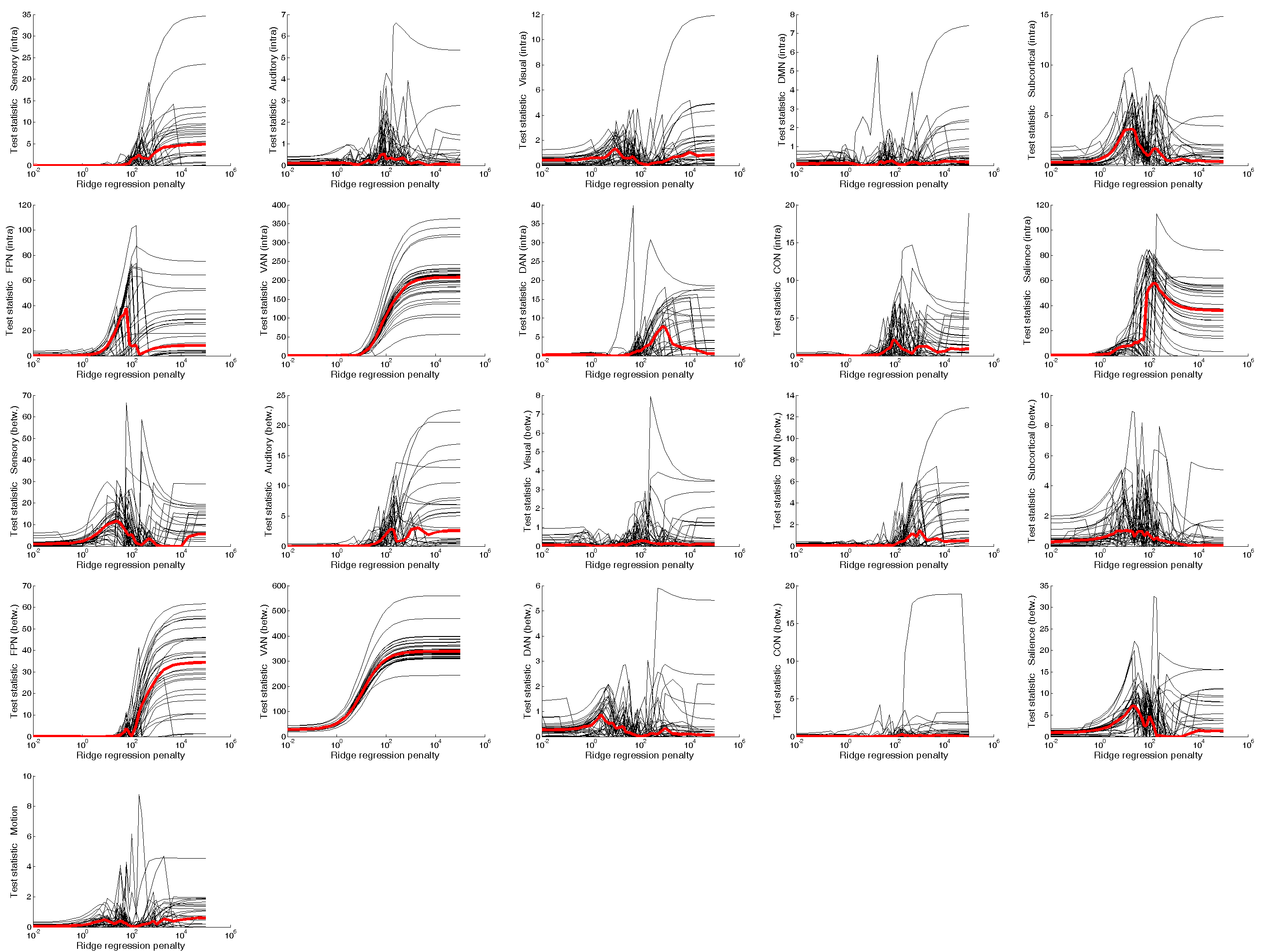


**Tables**

**Table S1.** Within- and between-system connectivity of each system

| **System** | **System Connectivity** | |
| --- | --- | --- |
|  | **Within** | **Between** |
| SM | 1.03 | 0.99 |
| Aud | 0.97 | 1.00 |
| Vis | 1.06 | 0.99 |
| DM | 1.03 | 1.00 |
| Sub | 1.00 | 0.98 |
| FP | 0.99 | 1.00 |
| VA | 0.92 | 1.00 |
| DA | 0.95 | 1.00 |
| CO | 1.00 | 1.01 |
| Sal | 0.99 | 1.00 |
| **Mean** | 0.99 | 1.00 |

**References**

1. Jenkinson M, Bannister P, Brady M, Smith S. Improved optimization for the robust and accurate linear registration and motion correction of brain images. *NeuroImage* 2002; **17**(2)**:** 825-841.

2. Satterthwaite TD, Elliott MA, Gerraty RT, Ruparel K, Loughead J, Calkins ME *et al.* An improved framework for confound regression and filtering for control of motion artifact in the preprocessing of resting-state functional connectivity data. *NeuroImage* 2013; **64**(0)**:** 240-256.

3. Hallquist MN, Hwang K, Luna B. The nuisance of nuisance regression: Spectral misspecification in a common approach to resting-state fMRI preprocessing reintroduces noise and obscures functional connectivity. *NeuroImage* 2013; **82C:** 208-225.

4. Greve DN, Fischl B. Accurate and robust brain image alignment using boundary-based registration. *NeuroImage* 2009; **48**(1)**:** 63-72.

5. Satterthwaite TD, Cook PA, Bruce SE, Conway C, Mikkelsen E, Satchell E *et al.* Dimensional depression severity in women with major depression and post-traumatic stress disorder correlates with fronto-amygdalar hypoconnectivty. *Molecular psychiatry* 2016; **21**(7)**:** 894-902.

6. Avants BB, Tustison NJ, Song G, Cook PA, Klein A, Gee JC. A reproducible evaluation of ANTs similarity metric performance in brain image registration. *NeuroImage* 2011; **54**(3)**:** 2033-2044.

7. Klein A, Andersson J, Ardekani BA, Ashburner J, Avants B, Chiang MC *et al.* Evaluation of 14 nonlinear deformation algorithms applied to human brain MRI registration. *NeuroImage* 2009; **46**(3)**:** 786-802.

8. Tustison NJ, Avants BB, Cook PA, Zheng Y, Egan A, Yushkevich PA *et al.* N4ITK: improved N3 bias correction. *IEEE Trans Med Imaging* 2010; **29**(6)**:** 1310-1320.

9. Tustison NJ, Cook PA, Klein A, Song G, Das SR, Duda JT *et al.* Large-scale evaluation of ANTs and FreeSurfer cortical thickness measurements. *NeuroImage* 2014; **99:** 166-179.

10. Power JD, Cohen AL, Nelson SM, Wig GS, Barnes KA, Church JA *et al.* Functional network organization of the human brain. *Neuron* 2011; **72**(4)**:** 665-678.

11. Muller K, Lohmann G, Neumann J, Grigutsch M, Mildner T, von Cramon DY. Investigating the wavelet coherence phase of the BOLD signal. *Journal of magnetic resonance imaging : JMRI* 2004; **20**(1)**:** 145-152.

12. Grinsted A, Moore JC, Jevrejeva S. Application of the cross wavelet transform and wavelet coherence to geophysical time series. *Nonlinear Proc Geoph* 2004; **11**(5-6)**:** 561-566.

13. White LB, Boashash B. Cross Spectral-Analysis of Nonstationary Processes. *Ieee T Inform Theory* 1990; **36**(4)**:** 830-835.

14. Bassett DS, Brown JA, Deshpande V, Carlson JM, Grafton ST. Conserved and variable architecture of human white matter connectivity. *NeuroImage* 2011; **54**(2)**:** 1262-1279.

15. Bassett DS, Porter MA, Wymbs NF, Grafton ST, Carlson JM, Mucha PJ. Robust detection of dynamic community structure in networks. *Chaos* 2013; **23**(1)**:** 013142.

16. Lockhart R, Taylor J, Tibshirani RJ, Tibshirani R. A Significance Test for the Lasso. *Annals of statistics* 2014; **42**(2)**:** 413-468.

17. Quenouille MH. Problems in Plane Sampling. *Ann Math Stat* 1949; **20**(3)**:** 355-375.

18. Quenouille MH. Notes on Bias in Estimation. *Biometrika* 1956; **43**(3-4)**:** 353-360.

19. Tukey JW. Bias and Confidence in Not-Quite Large Samples. *Ann Math Stat* 1958; **29**(2)**:** 614-614.
